# Intake of Fruits, Vegetables, and Sugar-Sweetened Beverages among a Sample of Children in Rural Northern Ontario, Canada

**DOI:** 10.3390/children9071028

**Published:** 2022-07-11

**Authors:** Brenton L. G. Button, Louise W. McEachern, Gina Martin, Jason A. Gilliland

**Affiliations:** 1 Human Environments Analysis Laboratory, Department of Geography and Environment, Western University, London, ON N6A 5C2, Canada; lmceach4@uwo.ca (L.W.M.); gmartin@athabascau.ca (G.M.); jgillila@uwo.ca (J.A.G.); 2 Faculty of Education, University of Winnipeg, Winnipeg, MB R3B 2E9, Canada; 3 Children’s Health Research Institute, London, ON N6C 2V5, Canada; 4 Faculty of Health Disciplines, Athabasca University, Athabasca, AB T9S 3A3, Canada; 5 Lawson Health Research Institute, London, ON N6A 4V2, Canada; 6 School of Health Studies, Western University, London, ON N6A 3K7, Canada; 7 Department of Paediatrics, Western University, London, ON N6A 5C1, Canada; 8 Department of Epidemiology & Biostatistics, Western University, London, ON N6A 5C1, Canada

**Keywords:** fruit and vegetable intake, sugar-sweetened beverages, rural youth health, rural heath, nutrition

## Abstract

There is evidence to suggest that dietary intake of children differs by rural/urban place of residence: rural children may have a higher intake of foods high in fat and sugar than those living in urban environments. The aim of this study was to examine the intake of fruits and vegetables (FV) and the frequency of sugar-sweetened beverage (SSB) consumption, among a sample of rural children in Northern Ontario, Canada, in two different seasons. Sociodemographic factors and children’s FV and SSB intake were measured using two repeated cross-sectional surveys, and seasonal information was based on the month of data collection. Logistic regressions were used to examine the odds of children eating five or more FVs, and the odds of ‘frequently or always’ consuming SSBs. During the fall, children reported eating five or more FV more often, when compared to winter (53.9% vs. 48.3%). In the fall, 25.8% of children reported ‘frequently or always’ drinking SSB, compared with 16.9% in winter. Indigenous children were less likely to eat five or more FV (OR 0.34 (95% CI 0.12–0.95)) in the fall when compared to non-Indigenous children. Findings indicate that intake of FV among rural students in this region is low, and the frequency of SSB is high, when compared with national recommendations.

## 1. Introduction

Evidence suggests that when compared to national guidelines diet quality among Canadian children aged 2 to 18 years is ‘poor’; many consume diets that are high in sugar, fats, and salt, and ones that are low in nutrient- and fibre-rich foods such as fruits and vegetables (FV) [1,2,3]. Poor-quality diets are linked to an increased risk of chronic disease [4], certain cancers [5], higher body mass indexes [6], and lower academic performance [7]. Dietary patterns established in childhood have also been shown to predict adult lifestyle-related disease [8], and thus, interventions aimed at youth have the potential to impact long-term health. Two specific groups of food that are of interest are FVs and sugar-sweetened beverages (SSB).

The 2019 version of Canada’s Food Guide recommends eating plenty of FVs each day and lowering consumption of SSBs by making water the drink of choice [9]. The previous version of Canada’s Food Guide (2007) recommended eating between six to eight servings of FVs per day for this age group [10], and the World Health Organization (WHO) recommends eating 400 g/day or approximately five servings a day [11]. However, only 56% of adolescents aged 12–19 years old reported consumption of at least five FVs per day in 2014 [12]. Canadian children aged 9–18 reported consuming an average of 300 ml/day of SSBs, equating to approximately 152 kcal/day [13], a considerable proportion of dietary energy intake for children of this age group [14].

It is critical to understand the determinants of a healthy diet to improve diet quality among Canadian children. Research shows that a child’s diet is influenced by factors such as age, gender, ethnicity, and parental education. Girls and younger children typically eat more FVs, but results are less conclusive for SSBs [15,16]. Higher parental education is related to higher FV consumption and lower parental education is associated with higher SSB consumption [16,17]. A further consideration in the Canadian context is that some areas have a higher percent of Indigenous peoples who report some differences in dietary intake when compared to non-Indigenous individuals [18]. However, a study by Riediger (2022) found no difference in dietary in-take between Indigenous and non-Indigenous individuals when food security status was controlled for [19].

There is also evidence to suggest that children’s dietary intake differs by rural/urban place of residence. Rural children in the USA were less likely to meet the recommendations for fruit intake and consumed on average 90 kcal/day more than their urban counterparts [20]; a similar pattern was noted among students attending rural schools in Alberta compared with those attending urban schools [21]. Conversely, a study comparing urban and rural children in Scotland found that rural children had the highest consumptions of FVs [22]. In a cross-Canada study among adolescents, rural students were more likely to report a higher frequency of consumption of SSBs than those living in urban environments [23]. These studies are limited as they focus on urban and rural differences rather than understanding determinants in rural areas, and these studies seem to suggest that the relationship between FV and SSB consumption may vary by place [20,21,22]. Living in a rural location can affect a child’s dietary intake as many rural areas have very few stores altogether, and of those stores, an extremely low number are actual, full-service grocery stores [24], which limits access to healthy foods [25].

Seasonal patterns can contribute to a child’s diet quality as the price and availability of FVs change throughout the year, particularly in rural areas. For example, in this area, in October, there are 8 fruits and 49 vegetables that are in season, compared to only 3 fruits and 17 vegetables in December [26]. A pattern of seasonal differences in FV consumption has been found in adults in Spain, with people eating more FVs in the summer [27]. It has also been suggested that socio-cultural traditions and environmental factors have an influence on food consumption, i.e., Thanksgiving, the hunting season, or Christmas holidays; the influence of marketing, i.e., back to school time; changes in ambient temperature, i.e., barbeque or outdoor cooking; other environmental stimuli, i.e., changing of leaves reminds people of the fall, and that can be associated with certain types of food [28]. With evidence showing a seasonal difference in food consumption, it has been recommended that public health interventions consider seasonal variation in consumption patterns [29,30].

While it is documented that these variables, i.e., age, gender, ethnicity, parental education, rurality, and seasonality, have an impact on FV and SSB consumption, how each of these determinants of health combines to create an environment that alters the intake of FV and SSB is understudied. With calls to action being made that reflect the unique make-up of rural communities, this study aims to answer the following questions [31]: 

What intake of FVs and what frequency of SSBs is reported by rural children?

Does reported consumption of FVs and frequency of intake of SSBs differ by season among rural children?

Are there factors that influence rural children’s consumption of FVs and SSBs, and do these differ by season?

With many rural children reporting lower self-rated and lower functional health than children in urban environments [32], this research is essential as it will inform policymakers, health units, and schools about dietary intake of rural children, and it will identify groups of children at higher risk of having an inadequate diet [31].

## 2. Study Context

This study uses data from the communities of Nipigon, Red Rock, Dorion, and the Lake Helen Reserve in Northern Ontario, Canada. These rural communities are located approximately 100 km (62 mi) from the nearest city of Thunder Bay, Ontario; populations of these communities range from 300 to 1650 people [33]. Two communities have a full-service grocery store, but they normally close at 6 pm and are sometimes closed on Sunday. For others, affordable access to healthy food is limited as the nearest grocery store is over a 30 min drive away. Having a grocery store that closes early or needing to travel long distances are common features of many rural and/or remote communities in Canada.

## 3. Materials and Methods

### 3.1. Study Design and Recruitment

Data for this study comes from the Spatial Temporal Environment and Activity Monitoring (STEAM) project. Full details can be found in other published work [34,35]. This study used a convenience sample, and all schools with grades 4–8 (approximately ages 8–13 years) in the four rural communities agreed to participate. Prior to informing the children, each school (principal or administrative staff) used their social media page to relay information about the study to the parents so they could either contact the research team or attend the in-school presentation and ask questions. At the in-school presentation, the research team gave a brief description to each class about the research study. At the conclusion of the presentation, a letter of information, a parent survey, and a parental consent form were given to each student to take home. To participate, each student had to return a signed parental consent form and provide their own assent. The first round of the STEAM project was conducted during late September and early October (2016). The second round began in late November and lasted to mid-December of the same school year, with the same students participating at these two different timepoints. Specific seasons were selected through consultation with school board principals and the ability to analyse seasonal differences in FV and SSB consumption. Principals requested these specific times so that basic results could be presented to student participants before graduation, and that results could be used to help with future school health planning.

**Figure 1:**
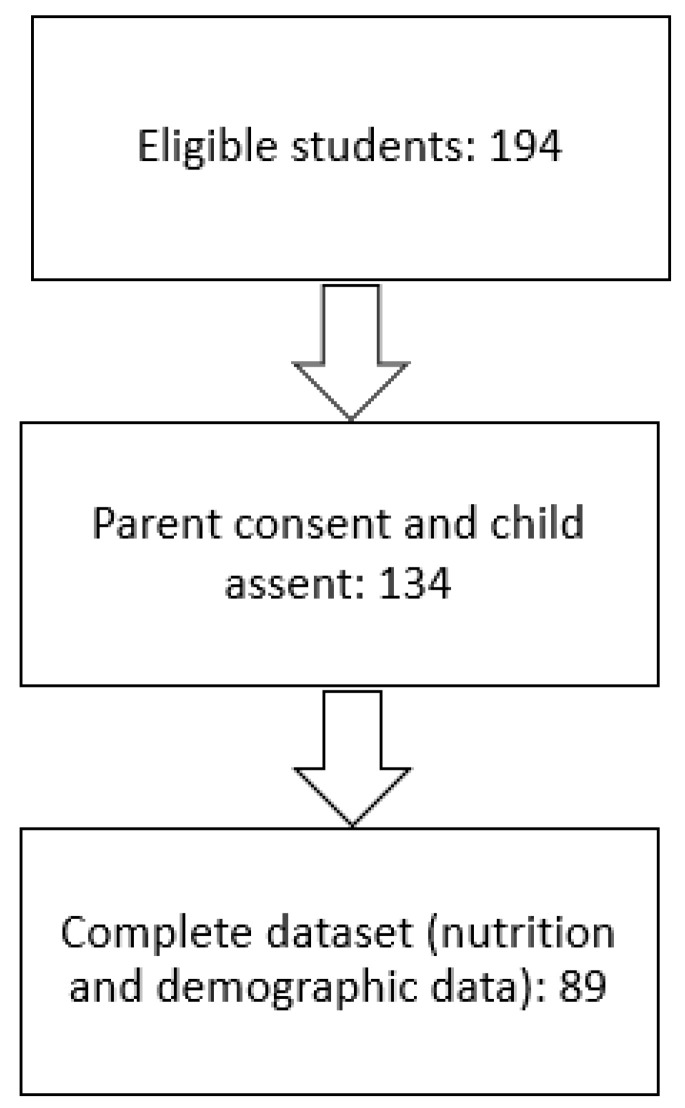
Flow chart from eligibility to final sample size.

Parent and child surveys collected data about demographic characteristics and consumption of FVs and SSBs (i.e., regular pop with sugar and fruit-flavoured drinks). Parent surveys were returned in a sealed envelope, and child surveys were conducted in class, with members of the research team responding to any questions to help maintain privacy. Of 194 eligible students, 134 had parental consent and provided their own assent to participate in this study. This sample was lowered further based on the following conditions: (a) the child had to have valid nutrition data in both the fall and winter; (b) both the child and/or parent filled out relevant demographic information on the child or parent survey. The final sample included 89 children; see Figure 1 for a flow chart. The sample had a similar breakdown based on age, gender, and ethnicity when compared to the excluded children. In this study, 28.1% identify as Indigenous compared to approximately 33% in the area [33]. The included participants were 58.4% female and had an average age of 10.5 years, compared to 57.6% female and an average age of 10.7 years in the excluded children.

### 3.2. Ethics

After consultation with school principals and a local First Nation band council ethics were submitted and approved by Western University’s non-medical research ethics board and the study was performed following the Declaration of Helsinki (NMREB File Number: 108029).

### 3.3. Independent Variables

Sociodemographic characteristics, including gender (boy/girl/other), age (continuous in years), ethnicity (White or Caucasian/South Asian/East Asian/Middle Eastern/Latin American/North American Indian, Metis or Inuit/Black, African, Caribbean), parental education (Grade 1–13/College or University/Graduate School/NA), were determined using self-administered surveys completed by both parent and child. Gender was dichotomized as boy/girl as no student responded any other gender identity in this sample. Ethnicity was dichotomized as non-Indigenous or Indigenous, as over 90% of participants self-identified as either Indigenous or White/Caucasian and previous research has shown differences between diets of Indigenous and non-Indigenous people. Parental education was dichotomized as high school or below/college and above based on distribution of the data and previous research that explored links between parental education and children’s diet [36]. All data came from the child survey except the question on parental education. Additionally, if a child did not answer a question on age, gender, or ethnicity, it was derived from the parent survey. The same survey tool was used in late September and early October and from late November to mid-December of the same school year. The fall season included data from September and early October, and the winter season had data from November to mid-December. In this region, during the first-time frame, temperatures are above freezing and include hunting season, Thanksgiving, and more in-season FVs. In the second time frame, temperatures are below freezing. Fewer FVs are in season, and roads may be closed due to inclement weather altering the accessibility of FVs. These variables were selected as the most essential based on previous literature and answering the specific research question as outlined in the Introduction.

### 3.4. FV and SSB Variables

The FV intakes were based on a previously validated FV screening measure [37], which asked, “In a typical day, about how many servings of fruit do you eat?” and “In a typical day, about how many servings of vegetable do you eat?” with none, 1, 2, 3, and 4+ listed as possible options. Examples for categories of food as well as serving sizes were provided (e.g., ‘one serving is equal to a piece of fresh fruit, like an apple or a small bowl of fruit salad’). No student reported 4+ in one category and 0 in another category. The scores from these two questions were added together and defined dichotomously as ‘below five’, or ‘five and above’. This aligns with the WHO, which suggests that children should eat roughly 400 g/day, which is approximately 5 servings [11].

SSB questions were based on survey questions developed by the STEAM team that used a 5-point Likert scale to gauge how often children consumed certain drinks. The SSB variable was derived by combining two questions that asked, “How often do you eat the following items?”, with regular pop with sugar and fruit-flavoured drinks listed ‘never’, ‘rarely’, ‘sometimes’, ‘frequently’, or ‘always’ as potential response options. Students that responded with ‘always’ or ‘frequently’ to either, or both, of these questions were considered to consume SSBs at a ‘high intake’, and all other students were used as the reference group representing children with ‘low intake’ of SSBs.

### 3.5. Data Analysis

All data analyses were performed in the Statistical Package for Social Science (SPSS) version 24 (IBM Corp., Armonk, NY, USA). Descriptive statistics, including means and frequencies, were used to describe sample characteristics, intake of FVs and frequency of consumption of SSBs. McNemar’s tests were performed on all children to examine associations between season and dietary variables, and logistic regression models were specified to examine the odds of children eating five or more FVs in the fall and winter, and the odds of consuming a high intake (‘frequently or always’) of SSBs in the fall and winter. A one-step method was used and considered gender, age, ethnicity, and parental education as covariates. A *p*-value of 0.05 was considered to indicate statistical significance.

## 4. Results

The final sample included 89 children. Table 1 presents the descriptive statistics for the sample. The mean age was 10.5 years, and there were more girls (58.4%) than boys (41.6%). During the fall, 53.9% of children reported eating five or more FVs per day and 48.3% reported eating five or more FVs per day in the winter (Table 2). During the fall, 25.8% reported a high intake (consuming ‘frequently or always’) of SSBs; 16.9% reported a high intake of SSBs in the winter (Table 2). Table 3 shows the results from McNemar’s test; no statistical significance was found when testing for an association between season and FV and SSB consumption.

No associations were found by children’s gender, children’s age, or parental education for either of the dietary variables examined within in each season (Table 4). Children’s ethnicity was associated with reported intake of FVs: the odds of eating five or more FVs were 0.34 (95% CI 0.12–0.95) times lower for Indigenous children in the fall, when compared to all other children (Table 4).

## 5. Discussion

The purpose of this study was to determine the number of servings of FVs and the frequency of SSB intake as reported by rural children aged 8–13 years from rural northern Ontario, to investigate if dietary intake of these foods/beverages differed by season, and to examine the variables that influence FV and SSB consumption in different seasons. Based on the data available, across both seasons, findings show that 51.1% of children reported consuming five or more FVs per day, and almost 20% of all children reported a frequent intake of SSBs. In the fall, ethnicity was associated with reported intake of FVs: Indigenous children in this sample appeared less likely to consume 5+ servings of FVs per day than all other children in this study. These results provide valuable information for rural researchers and health programmers.

This study found that around half of all children reported eating five or more FVs per day. These results support other studies indicating that the FV intake of school-aged children across Canada is generally below recommendations [1]. However, the proportion of children reporting consumption of 5+ FVs per day in our study is higher than the reported 39.5% for Canadians aged 12 and older [12], which is encouraging, as access to fresh FVs can be more challenging in many rural Canadian locations. A possible explanation for this finding is that each of the schools in the study area had a school breakfast programme that regularly provided FVs as part of their daily programmes. Some of these breakfast programmes are supported by the Northern Fruit and Vegetable Program [38]. School-based programmes are generally well liked and targeting the dietary intake of youth via this route is a popular approach; our results demonstrate the importance of continuing and investing in such programmes in rural areas. One way to increase the effectiveness of these programmes is by increasing variety of FVs offered [39].

Depending on the season, up to 25% of children reported a high intake of SSBs. This number could also be an underestimate as the study only asked about regular pop and fruit-flavoured drinks. Although “high” intake does not have a quantifiable measurement, this adds support to previous research in northern Ontario, which has suggested that a large proportion of youth regularly consume SSBs [40]; in general, SSB consumption among Canadian youth exceeds recommended levels [13,41]. This is a concern, not only from the point of view of sugar consumption, but also from the perspective of overall diet quality: a recent study has shown that compared with those who do not consume SSBs, high SSB-consumers eat more foods of a poorer quality [42]. Access to SSBs is convenient in terms of cost and availability, and these results add strength to efforts to reduce SSB consumption among youth at a local, provincial, and federal level [43,44]. However, commonly used policies such as SSB taxes must be examined with a health equity lens when considering implementation in remote, predominantly Indigenous communities, as many of these lack potable drinking water, pay exorbitant prices for milk, and are in essence forced to purchase SSBs as it is one of the only affordable options [45]. Future research is needed to explore what motivates youth to purchase SSBs in these rural and remote communities.

There was no association between season and FV consumption, but during the fall, children who self-identified as Indigenous were less likely to consume five or more FVs when compared to all other children. This result did reach statistical significance, but it should be interpreted with caution as multiple tests were run on the same data set. However, the result is plausible as previous research among Indigenous peoples has suggested a seasonal variation in nutritional profiles, explained by fluctuations in access to traditional food items [46,47]. A similar explanation is possible for this region; the fall data collection cycle aligned with hunting season for small birds, deer, and moose, and while traditional food intake was not assessed in this study, it is possible that Indigenous children had increased consumption of these foods during this time, taking the place of FVs eaten at other times of the year. Some small-scale changes including a community garden, a good food box, and a ‘Kids in the Kitchen’ programme have recently been implemented in response to higher rates of lifestyle-related chronic disease in Indigenous populations on the local reserve [48]. These programs can have myriad benefits including increased attachment to the land and food knowledge, strengthened social cohesion, and provide a safety net. Given the findings of this study, it is important for future research to evaluate these programmes to see if they have impact on the dietary intake of school children.

This study had some interesting null findings. Firstly, in comparison to research carried out in urban environments, gender did not appear to have an impact on FV consumption [15]. Research has suggested girls place a higher value on healthy eating when compared to boys [49], yet in general, rural areas tend to have lower levels of health literacy, which may not vary by gender [50]. In addition, parental education was not significantly associated with FV or frequency of SSB consumption. A study by van Ansem 2014 suggested that households with higher parental education as measured by maternal education had a greater availability of FVs and parents had a higher intake of FVs [51]. In rural areas, people have a more limited access to healthy and affordable food options because grocery stores of sufficient size selling an ample variety of affordable healthy foods require a significant amount of travel and time [52]. Thus, it can be difficult to access fresh and healthy foods in these regions regardless of parental education.

### Strengths and Limitations

While our study has a small sample size, which limits the statistical analysis and number of variables we can control for, we have included a high proportion of the school children who live in this rural region (436.8 km^2^). It is noteworthy that we had complete data for 89 elementary school children in this study, which is 45.9% of all the 194 children throughout the region who were enrolled in the eligible grade levels. This study is limited by the use of self-reported dietary data as it is vulnerable to recall bias, particularly in children, and might be less accurate when compared with objective measures. At the same time, the ambiguous ‘never, rarely, sometimes, frequently, and always’ could be interpreted differently by each student and could result in some measurement error. However, this study included children as young as 8 years old, so using more specific questions could also be misinterpreted and lead to measurement error. Finally, it is important to recognize that these results are not reflective of a full year since the data collection time-points were separated by just two months, but research has found differences in purchase patterns in as little as two months [53].

## 6. Conclusions

With rural areas being understudied and underrepresented in the academic literature, this study shows that FV intake, while at more encouraging levels than in some other regions, is below recommendations among these children, and the frequency of SSB intake is high. There were no differences in FV and SSB consumption between seasons, but ethnicity was important at varying points in the year. With rural areas having more limited healthcare resources than urban areas, this study provides health promoters with some targeted information on risk factors (i.e., ethnicity) which could be helpful in improving the diet quality of rural and remote children in Canada and other industrialized countries. With Canada having many unique areas with different defining characteristics (i.e., ethnicity, availability of resources), we argue that regional studies are necessary to further our understanding of different phenomenon and provide more targeted evidence.

## Figures and Tables

**Table 1:** Sociodemographic characteristics.

Variable	Number (%)
Gender	
Boys	37 (41.6)
Girls	52 (58.4)
Ethnicity	
Non-Indigenous	64 (71.9)
Indigenous	25 (28.1.)
Parental Education	
High School and below	18 (20.2)
College and above	71 (79.8)
**Variable**	**(Range/Mean (Standard Deviation))**
Age	8–13/10.5 (1.4)

Study characteristics for the 89 children included in the study.

**Table 2:** Fruits and vegetables (FV) and sugar-sweetened beverages (SSB) intake.

Dietary Variables	Number (%)
FV Intake–Fall	
Below five	41 (46.1)
Five or above	48 (53.9)
FV Intake–Winter	
Below five	46 (51.7)
Five or above	43 (48.3)
SSB Intake–Fall	
Low intake	66 (74.2)
High intake	23 (25.8)
SSB Intake–Winter	
Low intake	74 (83.1)
High intake	15 (16.9)

Descriptive statistics for FV and SSB intake.

**Table 3:** McNemar’s test for FV and season and SSB and season.

Dietary Variables	*p*-Value
FV Intake Fall vs. Winter	0.458
SSB Intake Fall vs. Winter	0.134

**Table 4:** Sociodemographic factors associated with FV and SSB consumption, during the fall and winter.

	Fall OR (95% CI)	*p*-Value	Winter OR (95% CI)	*p*-Value
Fruits and Vegetables (Five or above)				
Constant	1.03	0.99	0.74	0.86
Gender (*ref: female*)	1.78 (0.74–4.29)	0.20	0.53 (0.22–1.26)	0.15
Age	1.05 (0.77–1.43)	0.74	1.05 (0.78–1.42)	0.74
Ethnicity (*ref: non-Indigenous*)	0.34 (0.12–0.95)	**0.04**	1.29 (0.50–3.33)	0.60
Parental Education (*ref: high school or below*)	1.18 (0.40–3.51)	0.77	0.57 (0.19–1.67)	0.30
Nagelkerke R Square	0.09		0.05	
Sugar-Sweetened Beverages (High intake)				
Constant	0.49	0.71	6.04	0.44
Gender (*ref: female*)	0.45 (0.17–1.19)	0.11	0.56 (0.18–1.78)	0.32
Age	0.99 (0.71–1.40)	0.97	0.68 (0.45–1.05)	0.08
Ethnicity (*ref: non-Indigenous)*	1.12 (0.37–3.32)	0.85	2.82 (0.57–13.87)	0.20
Parental Education (*ref: high school or below*)	1.48 (0.47–4.63)	0.51	0.95 (0.22–4.04)	0.94
Nagelkerke R Square	0.05		0.12	

Italics indicate reference group. Values in boldface indicate statistical significance *p*-value < 0.05.

## Data Availability

The data presented in this study are available on reasonable request from the corresponding author. Data sharing: Data described in the manuscript, code book, and analytic code will be made available upon request pending ethics approval.
